# An Epileptic Girl With Erotomania Using Carbamazepine|Resistant to Treatment and Challenge of Starting Clozapine

**DOI:** 10.1002/ccr3.71741

**Published:** 2025-12-28

**Authors:** Fateme Roham, Shaghayegh Mohammadghasemi, Reza Bidaki, Iman Hasanabadi, Ahmad Shamsi

**Affiliations:** ^1^ Student's Research Committee AJA University of Medical Science Tehran Iran; ^2^ Department of Medical Laboratory Sciences, School of Paramedicine Alborz University of Medical Sciences Karaj Iran; ^3^ Department of Psychiatry, Research Center of Addiction and Behavioral Sciences, Non‐Communicable Diseases Research Institute Shahid Sadoughi University of Medical Sciences Yazd Iran; ^4^ Diabetes Research Center Shahid Sadoughi University of Medical Sciences Yazd Iran; ^5^ Student Research Committee Shahid Sadoughi University of Medical Sciences Yazd Iran

**Keywords:** carbamazepine, clozapine, epilepsy, Erotomania

## Abstract

The management of treatment‐resistant erotomania in patients with epilepsy requires a cautious pharmacological approach. This case demonstrates that replacing carbamazepine with valproic acid enabled the safe initiation of clozapine, resulting in resolution of erotomanic delusions while maintaining effective seizure control.

## 1 Introduction

Erotomania is a form of rare delusional disorder, which is also known as De Clerambault's syndrome [1]. This condition is marked by a delusional belief that one is romantically loved by another individual, typically someone of higher social standing [2]. The defining feature of Erotomania syndrome is the belief, typically held by a woman, that she is deeply loved by a man [2]. In this delusion, the man is perceived to have fallen in love first, made the initial advances, and is more in love with her than she is with him or she may not reciprocate the feelings at all [2]. The individual develops an elaborate delusional narrative surrounding this man's supposed affection, his pursuit of her, and her inability to escape his “romantic grip” [3]. The focus is entirely on being loved, not loving in return [2]. The woman is convinced the man can't find true happiness without her and, in cases where the man is married, dismisses the marriage as invalid [2]. Patients with Erotomania are often diagnosed with paranoid conditions such as paranoid schizophrenia or other forms of paranoid disorder [3]. This syndrome may last for several weeks to months in its recurrent form, eventually being replaced by a similar delusion, involving another man [3]. In its fixed form, the syndrome can persist for several years [3]. Erotomania is significantly more common in women, while its occurrence in men is rare and likely underdiagnosed [3]. Male Erotomania tends to appear more frequently in forensic settings, as male gender is considered a risk factor for violent behavior associated with this condition [2]. Erotomania requires proactive treatment and careful risk management [4]. Due to the complexity of the condition, a multifaceted treatment approach has been explored [4]. Antipsychotic medications have been found effective in reducing the intensity of delusions and managing associated behaviors [4]. In cases of secondary Erotomania, treatment primarily focuses on addressing underlying organic or psychiatric disorders [4]. Comprehensive management of both primary and secondary Erotomania involves a combination of pharmacological treatments, psychosocial interventions and risk management strategies [4]. Clinical experience suggests that monothematic delusions, such as Erotomania, tend to be particularly resistant to various treatment methods [4]. Recent studies highlight the advantages of second‐generation antipsychotics, such as risperidone and clozapine, which offer better tolerability compared to older medications like pimozide [4]. This improved tolerability may enhance patient adherence and overall treatment outcomes [4]. Clozapine is not recommended as an adjunct to carbamazepine therapy due to its increased risk of agranulocytosis [5]. In this case report we intended to present a case of Erotomania associated with epilepsy. This patient had been taking carbamazepine due to her epileptic condition, but her delusions had been resistant to many medications. Based on previous recommendations, we intended to initiate clozapine therapy. However, due to the interaction between clozapine and carbamazepine, we were compelled to switch carbamazepine to another medication, specifically valproic acid.

## 2 Case History/Examination

A 32‐year‐old single housewife woman with a high school diploma from a low socioeconomic class and poor appearance has presented. The patient's problem started a week before the visit. Symptoms include restlessness, insomnia, daytime sleepiness, decreased appetite, a depressed mood, self‐talking, lack of energy to do her work, no longer enjoying anything she used to enjoy, a firm belief that someone from a higher social class is in love with her, but she does not have visual or auditory hallucinations, nor suicidal thoughts.

The patient had a history of depression, restlessness, and erotomania delusions for 8 years. During these 8 years, the delusions were stable and there were no periods of absence of symptoms. She had a history of two hospitalizations in the psychiatric ward. In a previous hospitalization with symptoms of talkativeness, insomnia, restlessness, and irritability, she was diagnosed with bipolar disorder type 1 in the manic phase and received psychopharmacotherapy and electroconvulsive therapy (ECT) three times (Table 1). The treatment during the previous hospitalization was unsuccessful. The patient fell from a height at the age of three and then suffered from tonic–clonic seizure and was prescribed Carbamazepine. She has no history of substance abuse. Her sister and aunt have psychological disorders, but she is unaware of their disease. The patient is the youngest child in the family and, apart from seizures which were controlled with medication, had no history of other problems during infancy and childhood. The patient had no problems during education and had not been re‐examined. She was married 3 years ago, but they divorced after 6 months because of her disease. During childhood and adolescence, she had good interactions with her classmates and friends and has not been reclusive.

**TABLE 1:** Daily treatment and discharge prescription.

Drug name	1st day	7th day	12th day	Discharge prescription
Lithium carbonate	900 mg	900 mg	600 mg	600 mg
Risperidone	6 mg	6 mg	6 mg	4 mg
Clozapine	—	6.25 mg	50 mg	100 mg
Quetiapine	25 mg	25 mg	25 mg	25 mg
Escitalopram	5 mg	5 mg	5 mg	10 mg
Valproic acid	—	1000 mg	1000 mg	1000 mg
Carbamazepine	600 mg	300 mg	—	—
Clonazepam	—	—	—	1 mg

In the psychiatric evaluation, the patient did not appear well‐groomed, with hyperpigmented skin and acne vulgaris on the face, and she had partial cooperation. She doesn't have an attractive or beautiful face. The patient's psychomotor was agitated, and the mood was depressed and irritable.

Some differential diagnoses are considered: delusional disorder (erotomania subtype), schizophrenia, schizoaffective disorder, bipolar disorder type Ι with psychotic features, and another medical condition. In the end, the diagnosis of delusional disorder is confirmed.

The patient was hospitalized in the psychiatric ward for 12 days. Table 1 shows the daily treatment and discharge prescription. On the second day, the patient also received ECT, and a weekly complete blood count (CBC) was conducted on the sixth day.

Due to the patient's history of seizure, there was a challenge in discontinuing Carbamazepine. The dose of Carbamazepine was gradually reduced and then discontinued, after which valproic Acid and then clozapine was added in the treatment plan.

A week after starting clozapine, the patient's delusions resolved and no epileptic attack was observed (Table 1).

## 3 Methods (Differential Diagnosis, Investigations, and Treatment)

Psychiatric evaluations revealed delusion of love, ataxia, insomnia, self‐talking, and depressed mood. These findings were consistent with bipolar disorder type Ι, but did not entirely explain the full spectrum of her symptoms. In Raven Intelligence Quality test she scored lower limit normal and also she doesn't have a beautiful and attractive face.

The major differential diagnoses for erotomania were delirium neurocognitive disorder, but they are distinguished based on the chronology of symptoms, whereas delusions occur with mood episodes. It was excluded with accurate interventions, clinical and social history, and psychological symptoms. The patient was also evaluated for schizophrenia based on DSM‐5 criteria, which did not support the diagnosis.

## 4 Conclusion and Results (Outcome and Follow‐Up)

The management of this patient required a multidisciplinary approach. The treatment regimen focused on addressing both the neurologic symptoms of epilepsy and psychiatric symptoms associated with erotomania. Given the complexity of her presentation, a comprehensive diagnostic workup was initiated.

The patient's treatment regimen included valproic acid instead of carbamazepine (with dosage of 1000 mg at the end of Day 12) for controlling symptoms of epilepsy. It was due to prevention of the interactions. clozapine also was added in Day six for controlling the delusional symptoms of erotomania (6.25 mg daily and discharge prescription was 100 mg daily). Es citalopram was also required (5 mg daily). SSRIs help increase the levels of serotonin in the brain, which can improve mood and alleviate depression.

In addition to pharmacological treatments, nonpharmacological interventions were crucial for this patient. On the second day, the patient also received ECT.

Altogether, there were no special adverse or unanticipated events in the course of diagnostic and treatment procedures being reported. The patient's response to treatment was good after 2 weeks of treatment and there were no more delusions.

The patient underwent follow‐up evaluations and there are no more delusions of erotomania.

The result of this study indicates that replacement of Carbamazepine with sodium valproate in an epileptic patient simultaneously with clozapine dose adjustment is a safe and effective approach. This case upholds the antiepileptic effect of sodium valproate and prevents the interaction between clozapine and carbamazepine, whereas improvement symptoms of erotomania.

## 5 Discussion

Psychiatric and neurological disorders are common, and epilepsy is one of them. In this case, its occurrence at the same time as seizure requires more attention [6]. Furthermore, these two diseases may have common mechanisms and even influence each other. It is hypothesized that common causes in neurotransmitters, particularly in the temporal lobe and limbic system, have led to this phenomenon [6].

Carbamazepine is an antiepileptic drug commonly used to prevent seizure episodes in patients diagnosed with epilepsy [7]. As we said, clozapine is not recommended as an adjunct to carbamazepine therapy due to its increased risk of agranulocytosis [4]. But, clozapine can be more effective in the treatment of treatment‐resistant schizophrenia and other psychiatric disorders, and due to its effect on dopamine receptors, it is clear that it is effective in the management of delusional disorders, including erotomania [8]. Due to drug interactions between anticonvulsants and antipsychotics, discontinuation of carbamazepine is essential to minimize adverse interactions and improve the effectiveness of clozapine treatment [9]. At the first time that the patient was hospitalized, was under treatment with carbamazepine and other antipsychotic drugs; but there were no improvements. In the second time, we tapered carbamazepine—because of interactions—and started valproic acid. Meanwhile, we started clozapine. After 2 weeks, the patient responded well to the treatment and there are no more delusions of erotomania while following up.

In a study conducted by Chaudhury et al. in India, four cases of erotomania were examined. In all of these cases, there was improvement in biological functioning, but the delusions remained [10] (Table 2). Another study examined a 32‐year‐old woman with a history of epilepsy and symptoms of erotomania [11]. After 1 month of combined treatment with antipsychotic and antidepressant medications, she returned to normal and her erotomania delusions disappeared [11] (Table 2).

**TABLE 2:** Review of cases and treatments.

Author	Case	Drugs	Response to treatment
Sowmya et al. [10]	Woman 45 years old Married	Haloperidol	Mood improvement Unchanged delusions
	Woman 22 years old Unmarried	Valproic acid 250 mg Quetiapine 50 mg	Insisting on delusions
	Woman 37 years old Married	Olanzapine 20 mg	No delusions Insisting on beliefs
	Man 22 years old Unmarried	Olanzapine 10 mg	Mood improvement No hallucination unchanged delusions
Guillard et al. [11]	Woman 32 years old	Combined antipsychotic and antidepressive medication	Euthymic No delusions

The importance of multidisciplinary involvement in the management of patients with epilepsy and co‐occurring psychiatric disorders is highlighted. Likewise, it demonstrates the potential of clozapine in the treatment of irreversible erotomania.

## 6 Conclusion

This case underscores the importance of a comprehensive therapeutic approach in patients with overlapping neurological and psychological disorders. The co‐occurrence of epilepsy and erotomania presents a unique clinical challenge while the patient was using carbamazepine and we started clozapine for her. The importance of this case lies in the interaction between clozapine and certain antiepileptic drugs, as well as the fact that erotomania cases are often unresponsive to most medications; the significance of this case is therefore highlighted in case reports involving clozapine.

Regular neuropsychiatric evaluations and careful consideration of medication side effects and interactions are recommended for the patient.

## Author Contributions

**Fateme Roham:** writing – original draft, writing – review and editing. **Shaghayegh Mohammadghasemi:** writing – original draft. **Reza Bidaki:** conceptualization, data curation, supervision, writing – review and editing. **Iman Hasanabadi:** data curation. **Ahmad Shamsi:** writing – original draft.

## Funding

The authors have nothing to report.

## Consent

The authors certify that they have obtained written informed patient consent for publication. In the form the patient has given her consent for her clinical information to be reported in the journal. The patient understands that her name and initial will not be published and due efforts will be made to conceal her identity, but anonymity cannot be guaranteed.

## Conflicts of Interest

The authors declare no conflicts of interest.

## Data Availability

The data that support the findings of this study are available on request from the corresponding author. The data are not publicly available due to privacy and ethical restrictions.

## References

[1] H. W. Jordan , E. W. Lockert , M. Johnson‐Warren , et al., “Erotomania Revisited: Thirty‐Four Years Later,” Journal of the National Medical Association 98, no. 5 (2006): 787–793. 16749657 PMC2569288

[2] M. T. T. R. T. Valadas and L. E. A. Bravo , “De Clérambault's Syndrome Revisited: A Case Report of Erotomania in a Male,” BMC Psychiatry 20, no. 1 (2020): 516. 33097035 10.1186/s12888-020-02921-5 PMC7585286

[3] H. W. Jordan and G. Howe , “De Clerambault Syndrome (Erotomania): A Review and Case Presentation,” Journal of the National Medical Association 72, no. 10 (1980): 979–985. 6999163 PMC2552541

[4] N. Alotti , P. Osvath , T. Tenyi , and V. Voros , “Induced Erotomania by Online Romance Fraud—A Novel Form of de Clérambault's Syndrome,” BMC Psychiatry 24, no. 1 (2024): 218. 38509502 10.1186/s12888-024-05667-6 PMC10953121

[5] G. van Weringh , L. van Koolwijk , L. Haan , D. J. Touw , and M. B. de Koning , “Paralytic Ileus in a Patient on Clozapine Therapy Showing an Inverted Clozapine/Norclozapine Ratio After Switching Valproic Acid to Carbamazepine: A Case Report,” Therapeutic Advances in Psychopharmacology 14 (2024): 20451253241255487. 38827014 10.1177/20451253241255487 PMC11143807

[6] A. M. Kanner , “Management of Psychiatric and Neurological Comorbidities in Epilepsy,” Nature Reviews. Neurology 12, no. 2 (2016): 106–116. 26782334 10.1038/nrneurol.2015.243

[7] M. A. García , R. Cristofoletti , B. Abrahamsson , et al., “Biowaiver Monograph for Immediate‐Release Solid Oral Dosage Forms: Carbamazepine,” Journal of Pharmaceutical Sciences 110, no. 5 (2021): 1935–1947. 33610571 10.1016/j.xphs.2021.02.019

[8] H. Y. Meltzer , “New Trends in the Treatment of Schizophrenia,” CNS & Neurological Disorders Drug Targets 16, no. 8 (2017): 900–906. 28758583 10.2174/1871527316666170728165355

[9] C. E. Pippenger , “Clinically Significant Carbamazepine Drug Interactions: An Overview,” Epilepsia 28, no. Suppl 3 (1987): S71–S76. 10.1111/j.1528-1157.1987.tb05781.x 3319544

[10] A. V. Sowmya , N. Gupta , S. Dhamija , M. Samudra , S. Chaudhury , and D. Saldanha , “Erotomania: A Case Series,” Industrial Psychiatry Journal 30, no. Suppl 1 (2021): S249–s251. 34908701 10.4103/0972-6748.328821 PMC8611580

[11] V. Guillard , J. Mallet , and F. Limosin , “Erotomania and Mood Disorder: A Case Report and Literature Review,” European Psychiatry 33 (2016): S632.

